# Central action of FGF19 reduces hypothalamic AGRP/NPY neuron activity and improves glucose metabolism^★^

**DOI:** 10.1016/j.molmet.2013.10.002

**Published:** 2013-10-23

**Authors:** Geneviève Marcelin, Young-Hwan Jo, Xiaosong Li, Gary J. Schwartz, Ying Zhang, Nae J. Dun, Rong-Ming Lyu, Clémence Blouet, Jaw K. Chang, Streamson Chua

**Affiliations:** 1 Department of Medicine, Albert Einstein College of Medicine, Bronx, NY 10461, USA; 2 Department of Pharmacology, Temple University School of Medicine, Philadelphia, PA 19122, USA; 3 Phoenix Pharmaceuticals, Inc., Burlingame, CA 94010, USA; 4 Department of Pathophysiology, Kunming Medical University, Kunming, PR China

**Keywords:** FGF19, Diabetes, Obesity, AGRP/NPY neurons

## Abstract

Tight control of glucose excursions has been a long-standing goal of treatment for patients with type 2 diabetes mellitus in order to ameliorate the morbidity and mortality associated with hyperglycemia. Fibroblast growth factor (FGF) 19 is a hormone-like enterokine released postprandially that emerged as a potential therapeutic agent for metabolic disorders, including diabetes and obesity. Remarkably, FGF19 treatment has hypoglycemic actions that remain potent in models of genetic and acquired insulin resistance. Here, we provided evidence that the central nervous system responds to FGF19 administered in the periphery. Then, in two mouse models of insulin resistance, leptin-deficiency and high-fat diet feeding, third intra-cerebro-ventricular infusions of FGF19 improved glycemic status, reduced insulin resistance and potentiated insulin signaling in the periphery. In addition, our study highlights a new mechanism of central FGF19 action, involving the suppression of AGRP/NPY neuronal activity. Overall, our work unveils novel regulatory pathways induced by FGF19 that will be useful in the design of novel strategies to control diabetes in obesity.

## 1 Introduction

Fibroblast growth factor 19 (FGF19), and its mouse ortholog FGF15, is an ileum-derived enterokine, synthesized in response to postprandial release of bile acids [1,2]. Once absorbed by enterocytes, bile acids activate the farnesoid X receptor (FXR) which positively regulates FGF19 production and secretion into the bloodstream [1,2]. FGF19 was described initially for its role in the negative feedback loop regulating bile salt synthesis by the liver [3]. Recently, FGF19 has emerged as an important regulator of the postprandial adaptive metabolic response [4]. Indeed, after a meal, FGF19 stimulates glycogen and protein synthesis in the liver [4] as well as downregulates glucose production [5] in non-obese animals. In addition, *Fgf15* deletion results in glucose intolerance [4], while FGF19 transgenic mice displayed reduced glucose levels and improved insulin sensitivity [6]. Along these lines, *Fgf15* expression has been reported to be reduced in the intestine of diet-induced obese mice [7] and lower FGF19 serum levels were found in patients with type 2 diabetes [8]. However, the contributions of FGF19 (or FGF15 in mouse) to obesity and metabolic dysfunctions remain to be clarified [9].

While systemic treatment with FGF19 reduced weight gain and reversed diabetes in obese mice [10], hepatic responses to FGF19 have been shown to be impaired in obese mice [11] and patients with steatosis [12], suggesting that FGF19 may also act independently from the liver in these contexts. FGF receptor 4 (FGFR4) is believed to be the predominant receptor by which FGF19 mediates its hepatic effects [13]. Nevertheless, it is unlikely that FGF19 only interacts with FGFR4 as several reports have suggested that FGF19 can potentially activate all 4 FGF receptors in the presence of the co-receptor β-klotho [14–16]. Moreover, *Fgfr4*-null mice retained their ability to reduce glycemia after FGF19 treatment [17] and FGF19 analogs that are unable to bind to FGFR4 are able to improve glucose handling [18]. Thus, FGF19 could potentially interact with other receptors in other tissues to mediate its protective effects [19]. Supporting this concept, FGF19 is known to stimulate glucose transport in cultured adipocytes through FGFR1 [15]. In addition, FGF receptors are expressed in the brain (Brain Allen Atlas) [20] and central administration of FGF19 improved glucose homeostasis in lean rats [21]. Finally, an agonistic anti-FGFR1 antibody that mimics the blood glucose lowering ability of FGF19 [22,23] has been shown to accumulate in the median eminence of the hypothalamus after systemic injection in obese mice [22], suggesting that the brain could partly mediate FGF19 effects.

In this context, we wanted to assess whether central FGF19 signaling was effective to improve insulin-resistance and hyperglycemia in obese mice. We show that FGF19-induced signaling in the hypothalamus ameliorated glucose intolerance by improving insulin sensitivity. In addition, our findings support a mechanism by which FGF19 inhibits hypothalamic AGRP/NPY neurons.

## 2 Material and methods

### 2.1 Animals

Leptin-deficient (*ob*/*ob*, stock number 000632), Npy-hrGFP (006417), Pomc-eGFP (009593) and *A*^*y*^ (000021) mice were obtained from Jackson Laboratory and bred in house. *A*^*y*^ and *ob*/*ob* mice were maintained on Picolab chow 5053(minimum protein 20%, minimum fat 4.5%; PMI Nutrition International) and analysis were performed on 2 month-old *A*^*y*^ and 6 month-old *ob*/*ob* male. WT (C57BL6/J, stock number 000664), Npy-hrGFP and Pomc-eGFP mice were fed a high fat diet (60% of fat, Research Diet) starting at 4 week-old for 8–12 weeks. Mice were single-housed or group-housed in a temperature-controlled environment at 22–24 °C under a 12 h light/12 h dark cycle, water was provided *ad libitum*. All experimental protocols were approved by the Institute for Animal Studies of the Albert Einstein College of Medicine.

### 2.2 Intracerebral ventricular injection

For implantation of guide cannulas, mice were anesthetized with Ketamine and Xylazine. Custom 3.5 mm guide cannulas (Plastics One) were implanted into the third ventricle using a stereotaxic apparatus (coordinates from bregma: anteroposterior, −0.3 mm; dorsoventral, −3 mm). Animals were allowed to recover for at least a week. Then, the correct placement of the guides was verified by checking the feeding response to 5 μg/μl of peptide YY (Bachem) in aCSF [24]. Only mice with 5 fold increases of food intake in response to PYY were used for functional assays. For analysis of central FGF19 functions, 1 μl of vehicle (aCSF) or water-soluble FGF19 (2 μg per mouse, Phoenix Pharmaceuticals) was infused at a rate of 0.5 μl/min using a micropump and a custom 4.5 mm injector (Plastics One). When the 4-days icv injection protocol was used, icv injections were performed once a day (~4 h after the light onset). Mice had free access to food and water during the 4 days of treatment.

Mice acutely treated with FGF19 icv injection were fasted for 6 h and were treated twice with FGF19 (2 μg per mouse) or aCSF, when food was removed and 3 h apart. The ERK1/2 inhibitor U0126 (Calbiochem) was prepared as 10 μg/μl in 50% DMSO in aCSF. To assess the effects of U0126, the mice were fasted for 6 h and were treated twice with icv injection of U0126 (5 μg/0.5 μl per mouse)+aCSF, vehicle (50% DMSO in aCSF)+aCSF, U0126+FGF19 (2 μg per mouse) or vehicle+FGF19, when food was removed and 3 h apart. Vehicle and U0126 were delivered 30 min prior infusion of aCSF or FGF19.

### 2.3 Glucose and insulin tolerance tests

Obese mice that received the four daily icv injection (see detailed protocol above) were fasted and subjected to a glucose tolerance test (GTT) with intraperitoneal (ip) injection of glucose performed 3 h after the last icv injection of aCSF or FGF19. HFD-fed and *A*^*y*^ mice were fasted for 6 h and injected with 1 g of glucose/kg of body weight. Leptin-deficient males were fasted for 18 h and injected with 0.5 g of glucose/kg of body weight. Obese mice treated acutely with FGF19 or/and the ERK1/2 inhibitor U0126 (see detailed protocol above) were fasted for 6 h and injected with 2 g of glucose/kg of body weight, 2.5 h after the last icv injection. Insulin tolerance tests (ITT) were performed after 4 daily icv injections in mice fasted for 6 h. HFD-fed were injected with 0.5 U of insulin/kg of body weight while *A*^*y*^ and *ob*/*ob* mice received 3 U of insulin/kg of body weight.

Blood samples were collected from tail veins. Glycemia was determined using a glucometer (Abbot) and serum insulin levels were measured by ELISA (Linco Mouse Insulin kit).

### 2.4 Immunohistochemistry

Mice were anesthetized and perfused with heparinized phosphate buffered saline (PBS) followed by 4% paraformaldehyde in PBS. For single color staining, procedures were performed as previously described [25]. Briefly, sections were first incubated with a rabbit polyclonal antiserum directed against c-FOS (1:1000; Calbiochem Ab-5), washed, and incubated with a biotinylated secondary antibody (1:200; Vector Laboratories). Tissues were washed several times and incubated with ABC (Vector Laboratories) and developed in 0.05% diaminobenzidine (DAB)/0.001% H_2_O_2_ solution. For immunofluorescence staining of c-FOS or pERK1/2, sections were incubated with antibody against anti-c-FOS (1:1000, Calbiochem Ab-5) or with phospho-p44/42 MAPK (pERK1/2) (1:200; Cell Signaling, ref. 4370), for 48 h at 4 °C, and successively incubated with an anti-rabbit antibody conjugated to biotin (1/200, Vector Laboratories) for 2 h at room temperature (RT) and with Streptavidin-Cy3 (1/50, Invitrogen) for 3 h at RT.

### 2.5 Image analysis

Single staining of c-FOS positive nuclei (DAB staining) was visualized with a Zeiss Axiovision microscope. Native GFP and Cy3 fluorescence were visualized with appropriate lasers and emissions filters on a Leica SP2 AOBS confocal microscope (Analytical Imaging Facility, Albert Einstein College of Medicine). We evaluated the effects of FGF19 on neuronal populations located in the arcuate nucleus (Bregma −1.7 to −1.9 mm, coordinates in the brain atlas of Paxino and Franklin 2001). For each mouse, brain sections (*n*=3–4 per animal) were analyzed and a mean value for each animal was calculated.

### 2.6 Immunoblot analysis

Protein extracts were prepared as previously described [26]. Immunoblots were incubated with primary antibodies (Cell Signaling) against pERK1/2 (1/2000, ref. 4370), ERK (1/2000, ref. 9107), S473phospho AKT (1/2000, ref. 4060), pan AKT (1/1000, ref. 4691) and GAPDH (1/2000, ref. 5174). For AKT, membranes were first incubated with the anti-phosphoAKT and then stripped to quantify pan AKT.

### 2.7 Quantitative RT-PCR

Medio basal hypothalami (MBH) were rapidly dissected and frozen. RNAs were prepared using QiagenRNeasy tissue kit (Qiagen) and cDNAs were synthesized using SuperScript III and random hexamers (Invitrogen). Quantitative PCR was performed with the following primer sets: *Agrp* (forward: 5′-cggaggtgctagatccacaga-3′, reverse: 5′-aggactcgtgcagccttacac-3′), *Npy* (forward: 5′-atgctaggtaacaagcgaatgggg-3′, reverse: 5′-tgaaatcagtgtctcagggctgga-3′), and *β-actin* (forward: 5′-ctggagaagagctatgagctgcct-3′, reverse: 5′-ctcctgcttgctgatccacatctg-3′).

### 2.8 Statistical analysis

Data are shown as average±SEM. Unpaired Student *t* tests and one-way ANOVA tests were performed. Statistical analysis was performed with GraphPad Prism 5 and significance was accepted at *P*<0.05.

## 3 Results

### 3.1 Peripheral delivery of FGF19 activates ERK signaling in the hypothalamic arcuate nucleus

**Figure 1:**
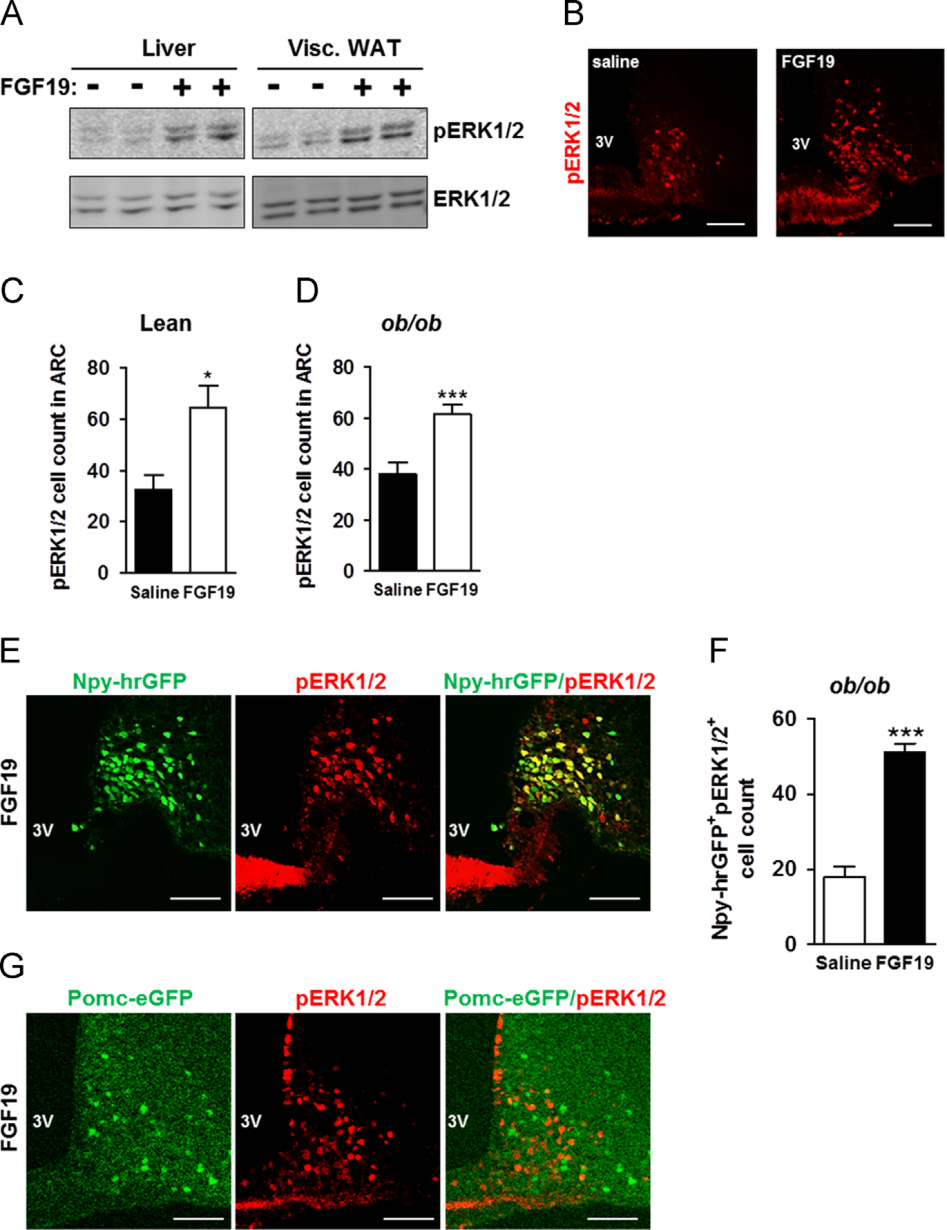
Intraperitoneal delivery of recombinant FGF19 promotes ERK1/2 activation in the arcuate nucleus of lean and obese mice. Levels of pERK1/2 were analyzed in response to intraperitoneal injection of FGF19 (0.5 μg/g of body weight) in WT lean mice or *ob*/*ob* mice fasted for 24 h. Mice were treated twice (starting 16 h after fasting and 6.5 h apart) and tissues were collected 90 min after the last ip injection. (A) Representative immunoblots of pERK1/2 and total ERK1/2 in liver and in visceral WAT of lean mice injected with saline or FGF19 (*n*=4 per group). (B) Immunofluorescence analysis showing pERK1/2 staining in the ARC of lean mice injected with saline or with FGF19. Quantification of pERK1/2 positive cells in ARC (Bregma −1.7 to −1.9 mm) of (C) lean mice (12 sections from 3 mice were used in each group) and (D) *ob*/*ob* mice (20 sections from 6 mice were examined in each group). (E) Analysis of co-localization between Npy-hrGFP positive neurons (green) and p-ERK1/2 positive cells (red) in Npy-hrGFP*ob*/*ob* mice fasted for 24 h. Mice were treated twice with ip injection of FGF19 (0.5 μg/g of BW, starting 16 h after fasting and 6.5 h apart) and tissues were collected 90 min after the last intraperitoneal injection. (F) Quantification of Npy-hrGFP positive neurons (green) that colocalized with pERK1/2 staining (red) in ARC of Npy-hrGFP*ob*/*ob* mice (10 sections from 3 mice were examined in each group). (G) Representative sections showing absence of co-localization between Pomc-eGFP positive neurons (green) and p-ERK1/2 positive cells (red) in Pomc-eGFP*ob*/*ob* mice fasted for 24 h and treated twice with FGF19 (0.5 μg/g of BW, starting 16 h after fasting and 6.5 h apart). Sections from 3 mice were examined in each group). ^*^P<0.05 and ^***^P<0.005. Scale bars: 100 μm.

Overexpression of FGF19 and intraperitoneal injection of recombinant FGF19 were previously shown to improve glucose metabolism in obese and diabetic mice [6,10]. *In vivo*, FGF19 can activate FGF receptor types 1 and 4 (FGFR1–4) and is known to stimulate ERK1/2 signaling in liver and adipose tissue [15]. Thus, to gain insight into the tissues targeted by intraperitoneal FGF19 injections, we assessed phospho-p44/42 MAPK (pERK1/2) levels in different tissues of lean mice. Mice fasted for 24 h were treated twice with 0.5 μg of FGF19 per gram of body weight. Ninety minutes after the last injection, tissues were collected for analysis of pERK1/2 level by western blot (liver and adipose tissue) or immunofluorescence (hypothalamus). In the liver and adipose tissue, we observed a significant increase in pERK1/2 (Figure 1A), as previously described [20]. We also examined the arcuate nucleus (ARC), the dorso-medial hypothalamus (DMH) and the ventro-medial hypothalamus (VMH). Interestingly, we found an increase in the count of pERK1/2 positive neurons in the ARC of lean mice (Figure 1B and C), while no pERK1/2 staining was observed in the DMH and the VMH (data not shown). Overall, these data suggest that at least the adipose tissue, liver and arcuate nucleus neurons can potentially mediate the glucose lowering effect of FGF19 in lean animals.

We next assessed whether the peripheral delivery of FGF19 was still able to induce ERK signaling in ARC of obese mice. As reported above for lean mice animals, a significant increase of pERK1/2 staining was observed in the ARC of leptin-deficient (*ob*/*ob*) mice after FGF19 treatment (Figure 1D).

Then, we investigated the neuronal population(s) targeted by FGF19 in obese animals. To answer that question, we first used Npy-hrGFP transgenic mice deficient in leptin (Npy-hrGFP*ob*/*ob*) fasted for 24 h, in which AGRP/NPY neurons in ARC appear GFP^+^. These mice were injected intraperitoneally with FGF19 and the colocalization between phospho-ERK1/2 and Npy-GFP was analyzed in ARC. We found that FGF19-induced pERK1/2 staining colocalized with Npy-GFP^+^ neurons (Figure 1E) and the count of Npy-GFP^+^ neurons co-expressing pERK1/2 was significantly increased after peripheral administration of FGF19 (Figure 1F). Similar experiments performed in Pomc-eGFP*ob*/*ob* mice showed that pERK1/2 staining did not colocalize with POMC neurons in ARC (Figure 1G). The lack of co-localization between POMC neurons and phospho-ERK1/2 staining unlikely comes from decreased *Pomc* expression resulting from leptin-deficiency, as such bias is mitigated by the use of accumulated enhanced GFP, rather than POMC peptides, to identify POMC neurons.

We then set out to examine whether FGF19 could signal in the hypothalamus to improve glucose homeostasis in obese insulin-resistant mice, as the ARC has emerged as a major center of convergence and integration of nutrient-related signals, critical in determining whole-body energy homeostasis [27,28].

### 3.2 Central delivery of FGF19 improves energy balance in obese mice

**Figure 2:**
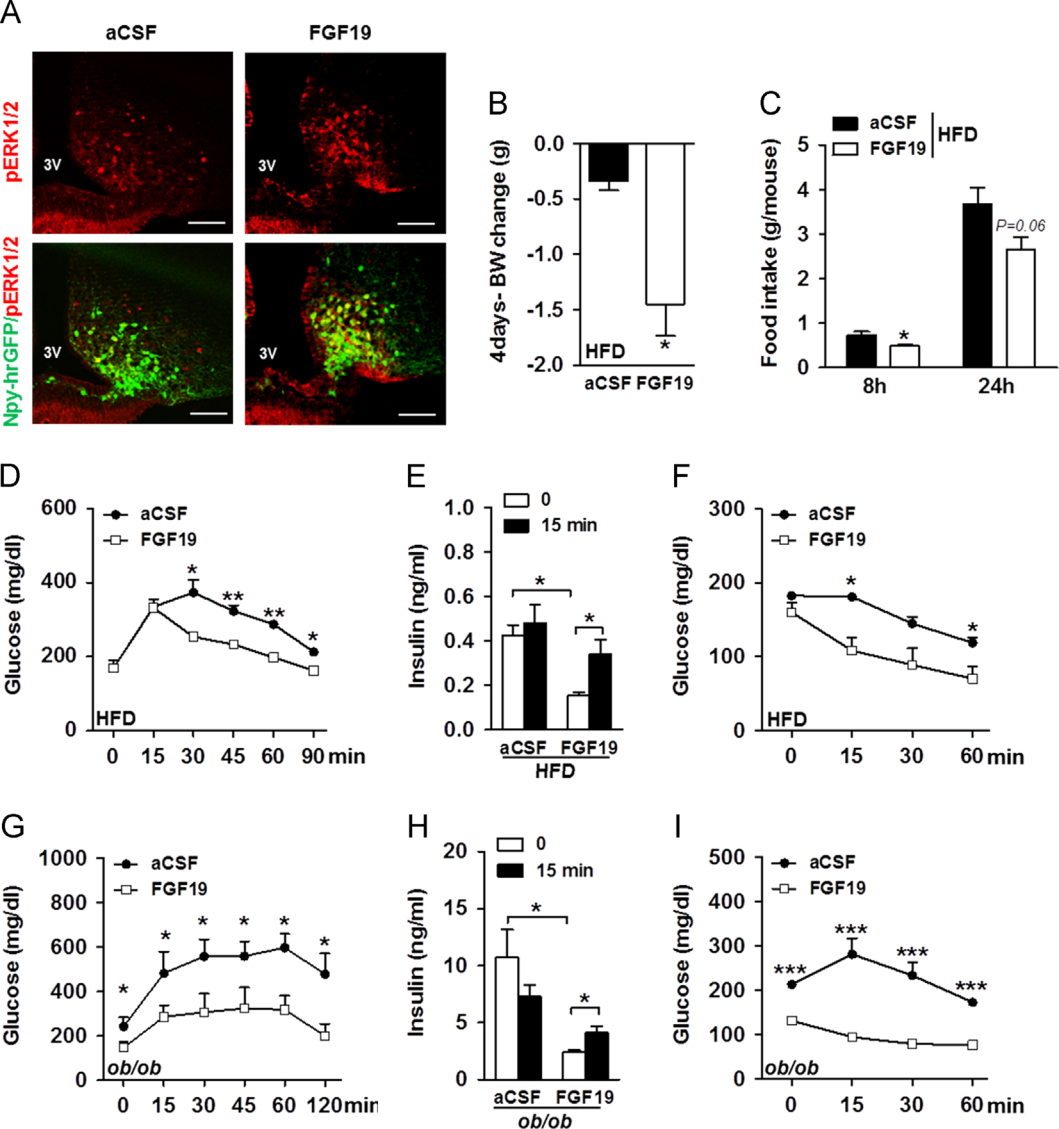
Central FGF19 delivery improves whole body energy balance in HFD-fed and *ob/ob* mice. (A) Representative sections showing pERK1/2 (red) and co-localization between Npy-GFP positive neurons (green) and p-ERK1/2 positive cells (red) in ARC of HFD-fed Npy-GFP males 90 min after icv injection of aCSF and FGF19 (*n*=3). (B) Body weight (BW) change of HFD-fed males after 4 daily icv injections of aCSF or FGF19 (*n*=9 per group). (C) Food intake per mouse was measured 8 and 24 h post-icv injection of aCSF or 2 μg of FGF19 in HFD-fed males (*n*=5 per group). (D) GTT, with intraperitoneal (ip) injection of 1 g of glucose/kg of body weight (BW), was performed on HFD-fed males, after 6 h of fast (*n*=4–5 per group). The last icv injection of aCSF or FGF19 was performed 3 h before the GTT assay. (E) Serum insulin concentrations were assessed before and 15 min into the GTT in HFD-fed males treated with 4 daily icv injections of aCSF or FGF19 (*n*=5 per group). (F) ITT was performed with 0.5 U of insulin/kg of BW in HFD-fed males treated with 4 daily icv injections of aCSF or FGF19, after 6 h of fast (*n*=4 per group).The last icv injection of aCSF or FGF19 was performed 3 h before the ITT assay. (G) GTT, with ip injection of 0.5 g of glucose/kg of BW, was performed on *ob*/*ob* males fasted for 18 h and 3 h after the fourth icv injection (*n*=6 per group). (H) Insulinemia was assessed before and 15 min into the GTT (*n*=6 per group). (I) ITT was performed with 3 U of insulin/kg of BW in *ob*/*ob* males, after 6 h of fast and 3 h after the fourth icv injection (*n*=4 per group). ^*^P<0.05, ^**^P<0.005, and ^***^P<0.0005. Scale bars: 100 μm.

In order to isolate the central effects of FGF19, we studied high fat diet (HFD) fed and *ob*/*ob* mice that received daily injections of FGF19 in the third ventricle for 4 days. As shown above with peripheral injection, third intra-cerebro-ventricular (icv) injections of FGF19 recapitulated ERK1/2 activation in arcuateNpy-GFP neurons of HFD-fed mice (Figure 2A).

We first analyzed body weight (BW) of HFD-fed mice treated with aCSF or FGF19, before and for the next 4 days after icv injection. Using a paired-analysis, we showed that body weight (BW) gain was significantly reduced in FGF19 treated HFD-fed mice (Figure 2B). This FGF19-induced BW loss could be due to reduced caloric intake as we measured decreased food intake in HFD-fed mice 8 h after a single icv injection of FGF19 as compared to aCSF treated animals (Figure 2C). Similarly, FGF19 treatment for 4 days reduced BW gain in *ob*/*ob* mice (+0.6±0.18 g and −0.25±0.15 g in mice treated with aCSF and FGF19, respectively; *P*=0.0043) which was associated with lower food intake 24 h after icv injection of FGF19 (5.02±0.97 g and 3.74±0.65 g in mice treated with aCSF and FGF19, respectively; *P*=0.0467). Consequently, daily icv of FGF19 suppressed body mass accretion as previously reported for daily intraperitoneal injection [10].

After 4 days of FGF19 icv injection, glucose metabolism was examined in HFD-fed mice and we found that basal fasting glycemia was unchanged after treatment (Figure 2D). Nonetheless, a glucose tolerance test (GTT) showed that central FGF19 injections markedly improved glucose tolerance in HFD-fed mice (Figure 2D). To better characterize the effects of central FGF19 signaling on glucose homeostasis, we measured insulinemia. Fasting plasma insulin level was reduced after FGF19 treatment (Figure 2E), suggesting increased insulin sensitivity. Accordingly, we performed insulin tolerance tests (ITT) and found that FGF19 markedly enhanced insulin-induced glucose lowering in HFD-fed mice (Figure 2F). Moreover, FGF19-treated mice displayed improved glucose-stimulated insulin secretion (GSIS) compared to aCSF-treated animals (Figure 2E), reflecting amelioration in pancreatic β cell function. Taken together, these findings demonstrate that delivery of FGF19 in the 3rd ventricle beneficially impacts on glucose metabolism and improves both glucose-induced insulin secretion and insulin sensitivity.

The same set of experiments was conducted on *ob*/*ob* animals. Contrasting with HFD-fed mice, fasting glycemia was significantly reduced in FGF19-treated *ob*/*ob* mice (Figure 2G). Amelioration of glucose tolerance was further confirmed in this model as central FGF19 injection improved glucose clearance during a GTT (Figure 2G). Similarly to HFD-fed mice, *ob*/*ob* mice treated with FGF19 had lower insulinemia (Figure 2H) that may be secondary to the increase in insulin sensitivity we observed (Figure 2I). In addition, we also found that GSIS was improved in FGF19-treated *ob*/*ob* mice (Figure 2H). All together, we conclude that central FGF19 ameliorated glucose homeostasis in HFD-fed and *ob*/*ob* mice, and is thus operative independently of leptin signaling. Regarding insulin secretion, even if the FGF19 treated mice appeared to be more responsive to glucose, insulin values at *t*=15 min were not different between aCSF and FGF19 treated mice. Therefore, the amelioration of glucose tolerance in FGF19 treated animals may be mostly attributable to higher insulin sensitivity rather than a consequence of insulin secretion.

**Figure 3:**
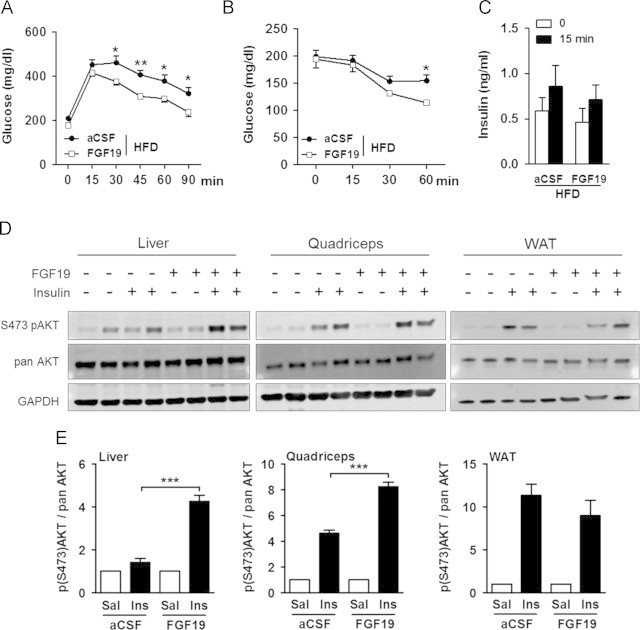
Acute icv administration of FGF19 improved insulin-signaling in HFD-fed mice. (A) GTT with 2 g of glucose/kg of body weight (intraperitoneal injection) and (B) ITT (0.5 U of insulin/kg of BW) were performed on HFD-fed males fasted for 6 h. (C) Serum insulin concentrations were assessed before and 15 min into the GTT in HFD-fed males treated with icv injections of aCSF or FGF19. Mice were treated twice with icv injection of aCSF or 2 μg of FGF19 (starting when food was removed and 3 h apart). Analysis were performed 3 h after the last icv (*n*=5 per group). (D) Ser473 phosphorylation of AKT, pan AKT and GAPDH expression in liver, quadriceps, and epidymal fat (WAT) of HFD-fed males. (E) Quantification of S473pAKT normalized against pan AKT in liver, quadriceps and WAT of HFD-fed males treated with icv injection of aCSF or FGF19. Mice were fasted for 18 h, treated twice with icv injection of aCSF or 2 μg of FGF19 (starting 12 h after fasting and 3 h apart) and received 8 U of insulin/kg of BW, 3 h after the last icv injection. Animals were sacrificed 8 min after insulin injection and tissues were collected for western blotting, (*n*=4 per group). Data for saline injections were arbitrary set to 1 and data after insulin injections were expressed relative to the value for saline injection for aCSF and FGF19 conditions. ^*^P<0.05, ^**^P<0.005, and ^***^P<0.0005.

Importantly, we wondered whether FGF19 glucose lowering effects were only attributable to body weight loss. To address that point, we studied the impact of acute FGF19 icv injection in HFD-fed animals and found that it improved glucose clearance during a GTT (Figure 3A) as well as increased insulin sensitivity (Figure 3B) in FGF19-treated mice while no difference in body weight was observed (−1.05±0.08 g *vs.* −0.94±0.09 g as BW change of mice 6 h after treatment with aCSF and FGF19, respectively; *P*=0.41). Analysis of insulinemia did not show any change between the aCSF and the FGF19 treated HFD-fed mice (Figure 3C), further confirming that amelioration of glucose tolerance by FGF19 may not rely on greater insulin secretion. We further investigated insulin signaling in HFD-fed mice and found that acute icv administration of FGF19 concomitantly to peripheral insulin injection markedly increased Ser473 phosphorylation of AKT in liver and skeletal muscle but not in white adipose tissue (Figure 3D and E). Thus, central delivery of FGF19 improved glucose metabolism and enhanced insulin signaling in HFD-fed mice independently of changes in body weight.

### 3.3 The impact of central FGF19 delivery on glucose metabolism relies on functional ERK1/2 signaling

**Figure 4:**
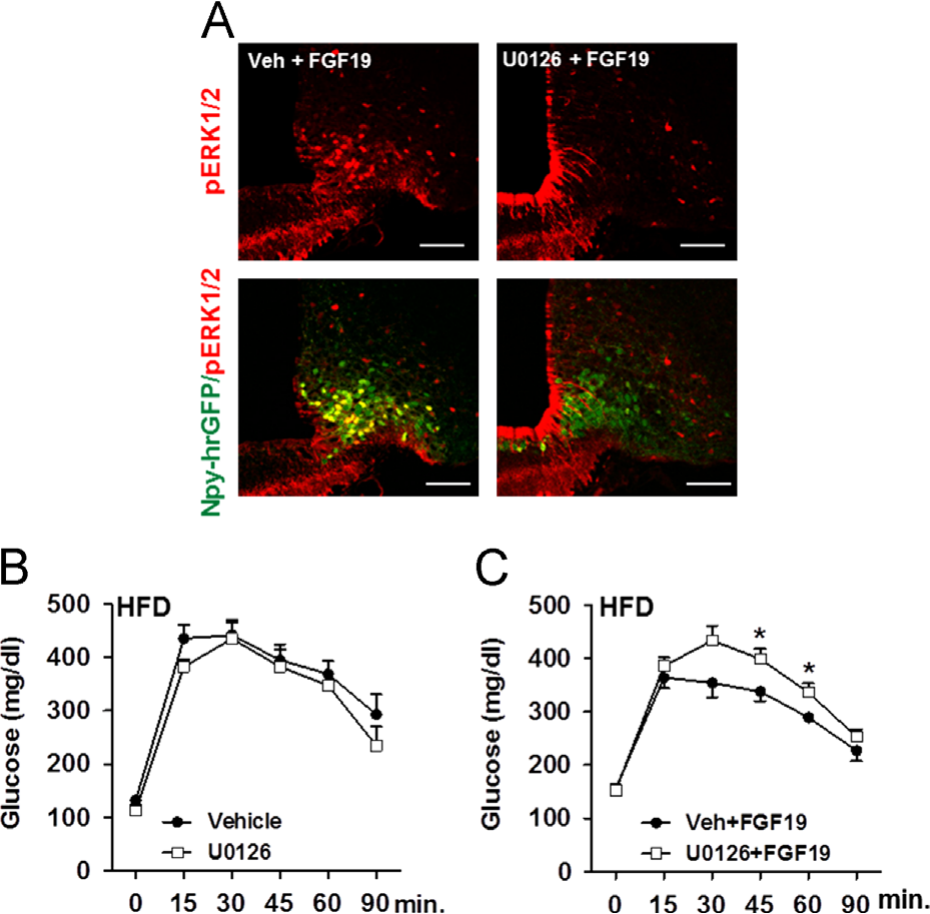
Central ERK1/2 signaling is involved in FGF19-mediated improvement in glucose metabolism. (A) Representative sections showing pERK1/2 (red) and co-localization between Npy-hrGFP positive neurons (green) and p-ERK1/2 positive cells (red) in ARC of HFD-fed Npy-hrGFP males 90 min after icv injection of vehicle+FGF19 and U0126+FGF19 (8–10 sections from 4 to 5 mice were examined in each group). (B) GTT with 2 g of glucose/kg of body weight (BW) was performed on HFD-fed males after 6 h of fast. Mice were treated twice with icv injection of vehicle+aCSF or U0126+aCSF (starting when food was removed and 3 h apart). GTT was performed 2.5 h after the last icv (*n*=4 per group). (C) GTT with 2 g of glucose/kg of BW was performed on HFD-fed males after 6 h of fast. Mice were treated twice with icv injection of vehicle+FGF19 or U0126+FGF19 (starting when food was removed and 3 h apart). GTT was performed 2.5 h after the last icv injection (*n*=8 per group). ^*^P<0.05 and ^***^P<0.005. Scale bars: 100 μm.

As FGF19 induced ERK1/2 activation in the ARC, we next assessed whether increased central ERK1/2 activation would mediate the glucose lowering effect of FGF19. To that aim, we delivered U0126 by icv injection in order to inhibit ERK1/2 phosphorylation in HFD-fed animals. Central injection of U0126 prior to icv administration of FGF19 blunted ERK1/2 phosphorylation in the ARC (Figure 4A). Importantly, we first determined that a dose of 5 μg per mouse of U0126 infused in the 3^rd^ ventricle of HFD-fed mice did not impact on glucose clearance during a GTT, excluding any effect of U0126 itself on glucose metabolism (Figure 4B). Then, we used a GTT for quantifying the effects of FGF19 to compare glucose tolerance between HFD-fed mice that were pre-treated with vehicle or with U0126 30 min before icv injection of FGF19. We found that pharmacological blockade of ERK1/2 phosphorylation dampened the glucose lowering effect of FGF19 during a GTT (Figure 4C). Overall, our data support that the gluco-regulatory effects of central FGF19 required activation of ERK1/2 signaling.

### 3.4 FGF19 repressed AGRP/NPY neurons activation

**Figure 5:**
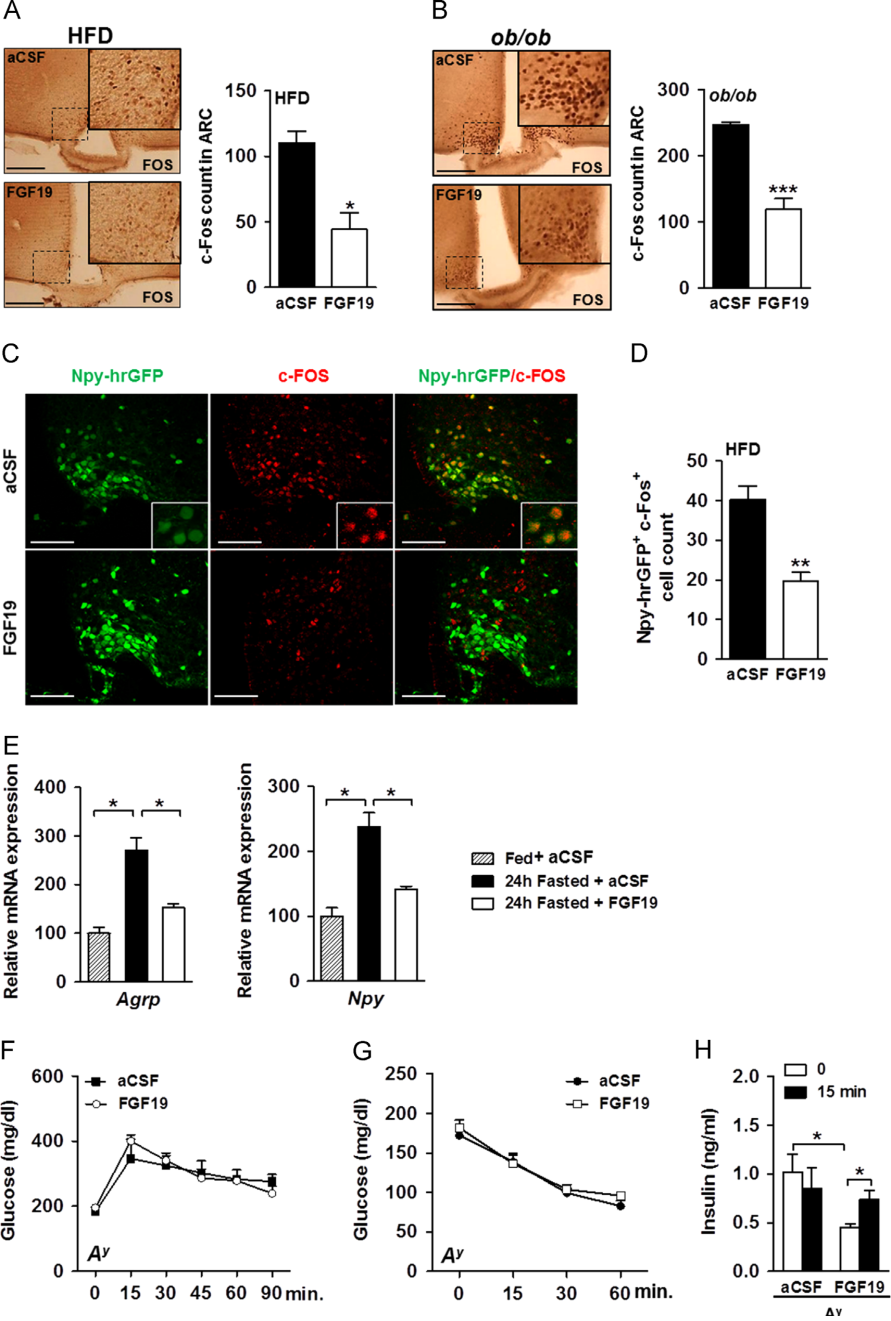
Central FGF19 inhibits fasting-induced activation of AGRP/NPY neurons. c-FOS immunostaining in fasted (A) HFD-fed and (B) *ob*/*ob* males, 90 min after the fourth FGF19 administration in the third ventricle (12 sections in ARC from 4 mice in each group were examined). (C) Analysis of co-localization between Npy-hrGFP positive neurons (green) and c-FOS positive cells (red) in HFD-fed Npy-hrGFP transgenic mice treated with icv injection of aCSF or FGF19 for 4 days. Animals were perfused 90 min after the last icv injection. (D) Quantification of Npy-hrGFP positive neurons (green) that colocalized with c-FOS staining (red) in ARC of HFD-fed Npy-hrGFP mice (9 sections in ARC from 3 mice in each group were examined). (E) Effect of FGF19 icv injection on *Agrp* and *Npy* gene expression in MBH of 24 h fasted wt mice treated with aCSF or FGF19 at the onset of fast and 12 h later. Fed and fasted mice were sacrificed 12 h after the last icv injection and MBH were collected for quantitative RT-PCR analysis. Data were *normalized* with -ctin expression level and *expressed* relative to the value for fed condition, which was arbitrarily *set* at *100*. (F) GTT with 1 g of glucose/kg of body weight (*n*=4 per group) and (G) ITT with 3 U of insulin/kg of body weight (*n*=5 per group) were performed on *A*^*y*^ males fasted for 6 h and that had received 4 daily icv injections of aCSF or FGF19. The last icv injection of aCSF or FGF19 was performed 3 h before the GTT and ITT assays. (H) Insulinemia was measured before and 15 min into the GTT in *A*^*y*^ males in aCSF and FGF19 treated group (*n*=4 per group). ^*^P<0.05, ^**^P<0.005, and ^***^P<0.0005. Scale bars: 100 μm.

We further examined the ability of FGF19 to modulate neuronal activity *in vivo* using immunohistochemical quantification of c-FOS expression, a marker of neuronal activation [29], in the hypothalamus of fasted HFD-fed and *ob*/*ob* mice. In both models, we observed a strong reduction in the number of c-FOS positive nuclei in ARC of FGF19-treated animals (Figure 5A and B) while similar c-FOS staining was found in the DMH and the VMH (data not shown), pointing to aninhibition of ARC neuronal activity following FGF19 icv administration.

AGRP/NPY neurons are known to be activated and to show increased c-FOS expression upon fasting [30,31]. To investigate whether FGF19 could suppress AGRP/NPY function by inhibiting their activation, we used HFD-fed Npy-hrGFP transgenic mice and found that central administration of FGF19 decreased the number of NPY-GFP^+^ neurons that were c-FOS^+^ (Figure 5C and D).

Then, we investigated the effects of FGF19 on *Agrp* and *Npy* gene expression in the medio-basal-hypothalamus (MBH) of mice fasted for 24 h and treated with icv injection of aCSF or FGF19. As expected, fasting induced expression of *Agrp* and *Npy* genes in MBH. However, FGF19 administration blocked the induction of *Agrp* and *Npy* expression (Figure 5E), further supporting a role for FGF19 in dampening AGRP/NPY neuronal activation. As AGRP/NPY neurons negatively regulate insulin action on glucose uptake and production [32], it is thus conceivable that FGF19-mediated repression of AGRP/NPY neurons activity improved glucose metabolism in obese mice.

### 3.5 The glucoregulatory effect of central FGF19 delivery is blunted in *A*^*y*^ diabetic mice

AGRP/NPY neurons activation is known to antagonize the central melanocortin signaling. Thus, we further investigated the implication of the melanocortin system in FGF19-mediated glucose lowering by assessing the impact of central FGF19 delivery in mice carrying the lethal yellow mutation (*A*^*y*^). *A*^*y*^ mice display ectopic expression of the normal agouti protein that constitutively antagonizes melanocortin receptors and leads to obesity, altered glucose homeostasis and insulin resistance [33,34].

Two months-old *A*^*y*^ males were hyperglycemic (132.3±9.61 mg/dl *vs.* 183.3±15.1 mg/dl, *P*=0.04 in WT and *A*^*y*^ males, respectively), hyperinsulinemic (0.36±0.12 ng/ml *vs.* 1.02±0.18 ng/ml, *P*=0.04 in WT and *A*^*y*^ males, respectively) and displayed glucose intolerance as compared to WT mice (6850±17.48 *vs.* 9136±187.5, *P*=0.0005, area under the GTT curve in WT and *A*^*y*^ mice, respectively). To assess the ability of central FGF19 to improve glucose intolerance in *A*^*y*^ mice, we used the same daily FGF19 icv injection protocol used above and that successfully improved glucose intolerance and insulin-resistance in HFD-fed and *ob*/*ob* mice. While a control group of *ob*/*ob* mice treated with icv injection of the same batch of FGF19 showed improved glucose homeostasis, as seen above, *A*^*y*^ mice displayed a blunted response to daily administration of FGF19. Indeed, both GTT (Figure 5F) and ITT (Figure 5G) curves remained unchanged after treatment, despite improved GSIS (Figure 5H). In addition, we saw no changes in BW (−1.43±0.46 g *vs.* −2.1±0.23 g as BW change of mice after a 4day-treatment with aCSF and FGF19, respectively; *P*=0.26) and in food intake (4.32±0.58 g *vs.* 4.22±0.47 g as 24 h-food intake following aCSF and FGF19 icv injection, respectively, *P*=0.89). Overall, our data support the contention that at least some of the glucoregulatory consequences of central FGF19 action rely on a functional MC3R/4 R signaling as FGF19 did not improve glucose handling in *A*^*y*^ mice.

## 4 Discussion

FGF19 has emerged as an important regulator of the postprandial adaptive metabolic response [4]. While effects of FGF19 on the liver have been described, another site of action such as the central nervous system (CNS) has also been suggested in the literature [21,23]. Here, we showed that peripheral delivery of FGF19 induced ERK1/2 activation in the adipose tissue and the liver but also the hypothalamic arcuate nucleus (ARC). This suggested that FGF19 might exert part of its glucose lowering effects through signaling in ARC neurons in lean and obese animals. This is compatible with the physiology of FGF19. Indeed, FGF19 is present in the bloodstream and recent reports demonstrated that neurons of the hypothalamic ARC have direct access to circulating concentrations of hormones and nutrients via the fenestrated capillaries perfusing this circumventricular organ [35,36].

In order to establish the role of central FGF19 signaling in glucose metabolism, we performed central infusion of FGF19 in the third ventricles of diabetic mice and thus characterized the effects of FGF19 that were specific to CNS targeting. We have shown here that central delivery of FGF19 reduced body weight gain in HFD-fed and *ob*/*ob* mice. Those effects were very similar to what was reported in a previous study with obese mice treated with daily intraperitoneal injection of FGF19 [10]. In addition, we found that this treatment was efficient in normalizing glucose metabolism in obese mice by improving glucose tolerance, insulin sensitivity and insulin secretion. Acute treatment with FGF19 in HFD fed mice, which did not impact on body weight, also improved glucose tolerance and enhanced insulin sensitivity in the liver and the skeletal muscle. However, insulin secretion was not modified following acute administration of FGF19. Consequently, in HFD fed mice, improved insulin secretion following central FGF19 injection may not be the primary effect leading to the global improvement of glucose homeostasis which, as we show, may rather depend on central FGF19 ability to potentiate insulin signaling in the periphery. In *ob*/*ob* mice, FGF19 glucose lowering effects has been shown to principally rely on insulin-independent glucose disposal but not on improved insulin sensitivity [37]. This discrepancy between the conclusions may come from the differences in the molecular mechanisms underlying the etiology of obesity in diet-induced obesity (DIO) and genetic obesity due to leptin-deficiency, differences in the timeline of the experimentations as well as differences in the methodologies [38]. However, the insulin-independent glucose disposal mechanism highlighted by Morton et al. may also take place in DIO. More investigations should be undertaken to evaluate the relative importance of insulin sensitivity and glucose effectiveness for FGF19 glucose-lowering effects in HFD fed mice.

As the glucose lowering effect of FGF19 could be separated from body weight change, our findings are reminiscent of the improved glycemic control observed in patients after gastric bypass surgery, which displayed improved insulin secretion and reduced insulin resistance, independently of weight loss [39,40]. Although gastric bypass surgery is being used as a treatment for type 2 diabetes, the mechanisms of action remain unclear [39]. The anatomical changes after gastric bypass surgery could lead to an increase availability of bile acids in the distal intestine [41], favoring the secretion of FGF19 as suggested by the increased plasma FGF19 concentrations observed after gastric banding [41]. In this context, our data may provide a mechanism for the improvement of glucose homeostasis after bariatric surgery.

By using U0126, which has been widely used to suppress activation of ERK and to examine the role of these enzymes, we found that the central effects of FGF19 relied on activation of ERK1/2 signaling. Interestingly, we showed that peripheral delivery of FGF19 triggered activation of ERK1/2 in AGRP/NPY neurons, as did central delivery. *Fgf* receptors were found to be expressed in hypothalamus [20] and AGRP/NPY neurons sorted from medio-basal-hypothalamus of Npy-hrGFP lean mice showed expression of *Fgfr1*, *Fgfr2* and *Fgfr3* but not *Fgfr4* (unpublished data), suggesting that FGF19 could directly modulate AGRP/NPY neuron activity. In addition, we found that central administration of FGF19 led to a decrease in c-FOS staining in AGRP/NPY neurons, indicating that FGF19 evoked a general inhibition of AGRP/NPY neuronal activity. Moreover, we showed that FGF19 prevented fasting*-*induced up-regulation of hypothalamic *Agrp and Npy* mRNA, further implying that it prevented AGRP/NPY neuron activation.

The central melanocortin system, which is localized in the hypothalamic arcuate nucleus (ARC) and consists of POMC and AGRP/NPY neurons, is known to play a major role in governing peripheral glucose homeostasis [32]. POMC neurons secrete α-melanocyte-stimulating hormone (α-MSH), an endogenous agonist of the melanocortin receptors (*i.e*. MC3R and MC4R in the CNS) that potentiates insulin action on glucose uptake and production [32]. By contrast, AGRP/NPY neurons have an opposite effect on glucose handling by producing AGRP, a specific competitive antagonist of MC3R and MC4R [32]. We can hypothesize that by blocking the inhibitory effects of AGRP/NPY neurons on melanocortin signaling, FGF19 delivery in the third ventricle may control glucose metabolism in insulin-resistant mice [28,32,42]. In agreement with this view, we found that glucose handling was not improved following central FGF19 infusion in *A*^*y*^ mice in which melanocortin receptors are constitutively blocked. By contrast, the glucose-lowering effect of FGF19 was found to be blunt but still detectable in *Mc4r*^−/−^ mice [37]. These contrasting effects could be due to the remained expression of functional MC3R in *Mc4r*^−/−^ mice. However, our results using the *A*^*y*^ mice were very surprising since the involvement of NPY and/or GABA, co-secreted neurotransmitters of AGRP, appeared to be insufficient to affect glucose handling. Because the use of the genetic models also present some limitations, such as developmental compensations that cannot be controlled, more experiments will be needed to evaluate the possible involvement of other pathway(s) in central FGF19 action.

Altogether, we provide evidence that FGF19 can signal in the hypothalamus to improve peripheral insulin signaling through induction of ERK1/2 signaling. In addition, our data support that some of the effects of FGF19 on energy metabolism involved the suppression of AGRP/NPY neurons activity. Interestingly, the “leptin-like” actions of central FGF19, still operative in leptin-deficient mice, would be useful to manage obesity and its associated defects.

## 5 Conclusions

The post-prandial enterokine FGF19 has been shown to alter carbohydrates metabolism in mouse models of obesity and diabetes. Although previous works had thoroughly documented that FGF19 targeted the liver to modify hepatic metabolism and maintain energy balance homeostasis in non-obese mice, specific target tissues were not completely identified [11]. Here, we provide evidence that FGF19 can signal in the hypothalamic arcuate nucleus to improve peripheral insulin signaling through induction of ERK1/2 signaling and suppression of AGRP/NPY neurons activity in obese and insulin-resistant states.

## Conflict of interest

The authors declare there are no conflicts of interest.
